# Sustainable materials selection with emerging structural materials

**DOI:** 10.1038/s44296-026-00099-7

**Published:** 2026-04-10

**Authors:** Sam Burdett, Mohit Arora, Rupert J. Myers

**Affiliations:** 1 Department of Civil and Environmental Engineering, Imperial College London, South Kensington Campus, London, UK; 2 Skanska UK, 1 Hercules Way, Leavesden, UK; 3 Net Zero Centre, Department of Engineering, King’s College London, Strand, London, UK

**Keywords:** Engineering, Environmental sciences, Materials science

## Abstract

The construction industry is rapidly changing to meet growing demand and reduce its environmental impact. These objectives can be met, in part, through improved selection of construction materials. However, the material properties including embodied carbon (EC) of emerging construction materials are less well documented in material property databases compared to conventional ones, providing barriers to their utilisation and correct perception of their decarbonisation potentials. This study provides material property data for emerging structural materials through a comprehensive literature review, visualises the results on material property charts, and analyses these data comparing to conventional materials. Only 18% (37 out of 204) of the emerging structural materials reviewed had EC values; less (11%) had embodied energy values. Analysis of the data demonstrates that using alternative and emerging materials for structural beams and columns can substantially reduce EC. For example, in the beam case study presented and using cradle-to-gate EC data (excluding stored carbon in wood), engineered wood products (glulam, cross-laminated timber) and reused steel achieve 3-5% of the EC of primary steel. Therefore, we highlight the benefits of collecting material property and environmental impact data for emerging materials and their potential for greater adoption to achieve lower carbon construction.

## Introduction

The quantity of materials extracted from Earth annually has almost quadrupled since 1970 and totalled over 106 billion tonnes in 2024^1^. The construction sector accounts for approximately half of global annual materials demand by mass^2,3^. These materials are used to construct, maintain, and refurbish the built environment, e.g., buildings and infrastructure. Across the whole life of buildings and infrastructure, material use has a significant environmental impact. For newly constructed office, warehouse, and residential buildings in the United Kingdom (UK), it is estimated that between 67 and 76% of their whole life cycle CO_2_-eq. (‘carbon’) emissions are ‘embodied’, a significant proportion of which arise from materials production^4^.

The life-cycle carbon emissions of a building may be separated into ‘operational’ and ‘embodied’ components. Operational carbon emissions are those from electricity, gas or fuels used to operate building services (e.g., lighting, heating, ventilation, and other equipment)^5^. As a result of shifting towards renewable energy, technology and efficiency improvements, and more stringent energy regulations (e.g., in the UK and Europe), operational emissions have fallen in recent years and are expected to decline further in the future^6^. This trend is becoming globally relevant via an increasing focus on net-zero energy buildings, where operational energy requirements are minimised and/or met with increasingly renewable sources. Therefore, the importance of reducing material production emissions has increased in prominence, with the recognition that lower-carbon footprint alternatives are needed. Continued use of business-as-usual materials and methods risks overshooting national carbon budgets consistent with 1.5 °C–2 °C temperature rise relative to pre-industrial levels (1850–1900)^7^.

The embodied carbon (EC) emissions, or global warming potential (GWP) (kg CO2-eq), of buildings and infrastructure are those that are needed to bring them into existence, and are emitted throughout their life cycles, from raw material extraction, through production, use, and end-of-life^8^. This includes emissions associated with processing, e.g., fuel used in transport and/or energy used in product manufacture and packaging. EC is a widely adopted metric for assessing the environmental impact of buildings and infrastructure and for benchmarking construction sustainability.

In terms of the EC of buildings and infrastructure, structural materials (and components) are particularly important given that within newly constructed buildings they present the greatest contribution towards EC (between 46 to 67% for the superstructure and substructure) and thus offer the greatest potential for reducing EC^9–11^. Structural materials are those that have a long life and bear the load of the structure (dead load) and resist loading conditions such as occupants or wind (live load). Non-structural and finishing materials are those whose primary function is not load bearing but instead are used to ensure airtightness, reduce heat loss, prevent fire spread, or for aesthetic reasons. These typically contribute around one-third (37% for the internal finishes and façade) of the EC for a school building and less for other building typologies, e.g., commercial and residential^10^.

Novel structural materials are being developed that offer equivalent or improved functional performance while reducing environmental impact. Traditional construction materials can be substituted for novel, high-performance alternatives that, for example, can absorb CO_2_ within their structure or extend the range of material properties. New materials such as ultra-high-performance concrete exhibit very high compressive strength (greater than 200 MPa), ductility (due to the addition of metal fibres), and durability^12,13^. High strength steels that exhibit characteristic 0.2% proof stresses of 960 MPa (2.7x greater than typical structural steels) have been characterised^14^. Therefore, more slender structural components are possible, enabling material efficiency and, in turn, reduction of EC. More generally, materials that offer an increase in property values can offer the same performance at lower material demand, generally increasing material efficiency within the built environment. The push for these materials is global and partly driven by regulatory pressure (specifying the measurement of and limits on EC), and partly by the interests of stakeholders in reducing EC. Examples include the AB-2446 EC Emissions: Construction Materials bill passed in California^15^, and mandatory life cycle assessment (LCA) measurements on buildings over 1,000 m^2^ in Denmark from 2023 onwards (Danish Building Regulation BR18). Given the momentum towards such regulations that aim to deliver a net-zero construction sector, the availability of lower EC materials is crucial for designers, architects, and structural engineers.

Irrespective of innovations and advancements in alternative material production, the lack of detailed consolidated information (e.g., for material properties) remains a key challenge. Even though several material property databases exist, which bring together standardised data on the physical, mechanical, thermal, environmental, and lifecycle attributes of materials, their lack of information on a comprehensive range of structural materials limits the opportunities for their adoption in the construction sector. These databases, such as the Inventory of Carbon and Energy (ICE) or Ansys® Granta EduPack, are primarily used by architects, structural engineers, materials scientists, and sustainability consultants during the early stages of building design and retrofit projects. They are used when multi-criteria decision-making is required, such as balancing performance and environmental impact, or comparing traditional materials with emerging alternatives. Although ad-hoc comparisons can be made using disparate sources, databases enable efficient, systematic analyses, and are often integrated with Building Information Modelling (BIM) tools. These tools are particularly useful in large-scale projects where manual data aggregation would be time-intensive and prone to inconsistencies.

Generally, these existing databases lack information on emerging materials e.g., as of December 2022, the only engineered wood entry in *Ansys® Granta EduPack v2022R2* was glue laminated timber (glulam), despite many engineered wood products currently being available, including cross laminated timber (CLT), dowel (mechanically) laminated timber, structural composite timber, bamboo (glulam), and glulam or CLT containing secondary (recycled) timber.

Even in cases where some material information is available, the potential contribution of emerging materials to reducing the overall EC of buildings remains unclear. To clarify this potential, material property data on emerging materials must be combined with traditional materials in materials property databases to allow for comparison during materials selection and design. In that pursuit, this study focuses on three broad research questions:

1. What is the current state of emerging structural materials as represented in existing databases?
2. What data are available on material properties and EC for emerging materials, and does this extend the materials property space?
3. How can material property and environmental data be integrated into a singular database that enables materials selection with low embodied emission material candidates?

To address these questions, this study develops a unified database of traditional and emerging construction materials including material property and environmental impact data, and analyses their differences using existing datasets (e.g., *Ansys® Granta EduPack v2022R2*^16^, Inventory of Carbon and Energy database 3.0)^17^.

The main novelty of this paper is its critical analysis of the relative properties and performance of emerging structural materials, compared to conventional structural materials. Taking a critical review approach, we develop Ashby-style charts to visually assist this comparison, to screen materials for specific applications, and to highlight where advancements in material properties have been made by emerging materials. The critical analysis is further enabled through our compilation of a large range of disparate material property data for emerging structural materials, which is a secondary novel aspect of the paper.

We prioritise structural materials, given they offer greater potential to reduce EC of buildings than non-structural materials, with their material property data provided in Supplementary Dataset 1. Additionally, we explore use cases of materials that serve specific functions (e.g., structural elements) to draw conclusions about the functional performance of the emerging materials within the dataset and demonstrate its utility in future research and practice.

## Results

We developed a dataset representing 409 materials across metals and alloys (*n* = 59), polymers and elastomers (*n* = 3), hybrids (*n* = 157), and ceramics and glasses (*n* = 197) (Table 1). The number of datapoints for a specific material is typically greater than the total presented below given that individual sources often contained multiple datapoints. We categorised these materials into ‘emerging’ and ‘traditional’ based on their adoption across the construction industry, with ~50% of the data collected here belonging to each material class.

**Table 1:** Total number of dataset entries collected in this study ordered by family, class and materials

Family	Total	Class	Total	Material	Total
Metals and alloys	59	Ferrous alloys	51	Coated steel	2
				Low alloy steel	2
				Low carbon steel	41
				Stainless steel	6
		Non-ferrous alloys	1	Aluminium	1
Polymers and elastomers	3	Thermoplastics	3	Synthetic low-density aggregate	3
Hybrids	157	Composites	106	Natural material	89
				Natural/synthetic	12
				Polymer	5
		Natural materials	51	Wood	32
				Engineered mycelium products	19
Ceramics and glasses	197	Nontechnical ceramics	197	Cement	13
				Cement paste / mortar	22
				Concrete	117
				Fired mineral	8
				Unfired mineral	15
				High density aggregate	13
				Low density aggregate	9

For all the materials shown in Table 1, we collected data on the following 21 material properties shown in Table 2. There was a primary focus on structural properties. Values in parentheses represent the units given in SI base units.

**Table 2:** Material properties included in the dataset

Material Property	Unit
Compressive strength, f_c_	Pa (N⋅m^−2^ or kg⋅m^−1^⋅s^−2^)
CO_2_ uptake	%
Density, *ρ*	kg⋅m^−3^
Freeze-thaw	% disintegration
Embodied carbon for 1 kg of structural material	kg CO_2_-eq⋅kg^−1^
Global warming potential, GWP	kg CO_2_-eq
Modulus of elasticity (MOE) / Young’s modulus (E)	Pa (N⋅m^−2^ or kg⋅m^−1^⋅s^−2^)
Modulus of rupture (MOR) / Flexural strength	Pa (N⋅m^−2^ or kg⋅m^−1^⋅s^−2^)
Moisture content, MC	%
pH	-
Plastic strain at fracture	-
Sound absorption coefficient	%
Shear strength	Pa (N⋅m^−2^ or kg⋅m^−1^⋅s^−2^)
Strain at ultimate tensile stress	-
Tensile strength, s_t_	Pa (N⋅m^−2^ or kg⋅m^−1^⋅s^−2^)
Ultimate bending moment	N⋅m (kg⋅m^2^⋅s^−2^)
Ultimate load	N (kg⋅m⋅s^−2^)
Upper yield strength	Pa (N⋅m^−2^ or kg⋅m^−1^⋅s^−2^)
Water absorption	%
Weight apparent sound reduction index	dB
Yielding load	N (kg⋅m⋅s^−2^)

### Young’s modulus, E

**Fig. 1:**
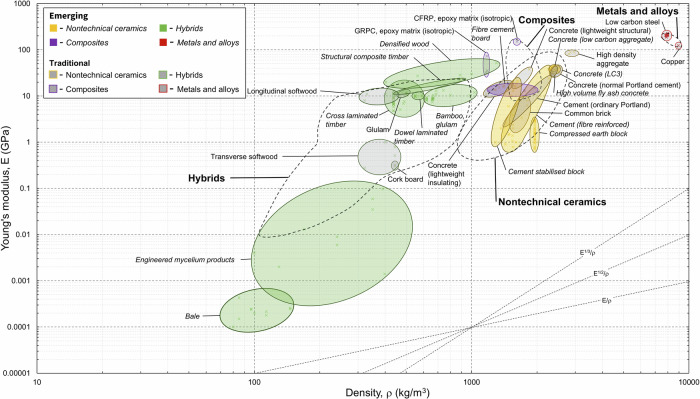
Materials property chart for Young’s modulus (E) with respect to density (ρ). The labelled ovals enclose material property data collected for this study, with all datapoints plotted at 50% transparency. Ovals filled in grey enclose material property data for traditional materials and ovals filled with colour (yellow, green, purple) enclose material property data for emerging materials. The dashed lines encompass material properties for conventional materials from a given material family from EduPack. Ovals plotted outside (or partially outside) of their respective material family extend the current materials property space. Material index guidelines are included in the bottom right-hand corner. The use of these lines is discussed in the section titled ‘*Materials Selection Using Material Indices and Case Studies’*.

**Fig. 2:**
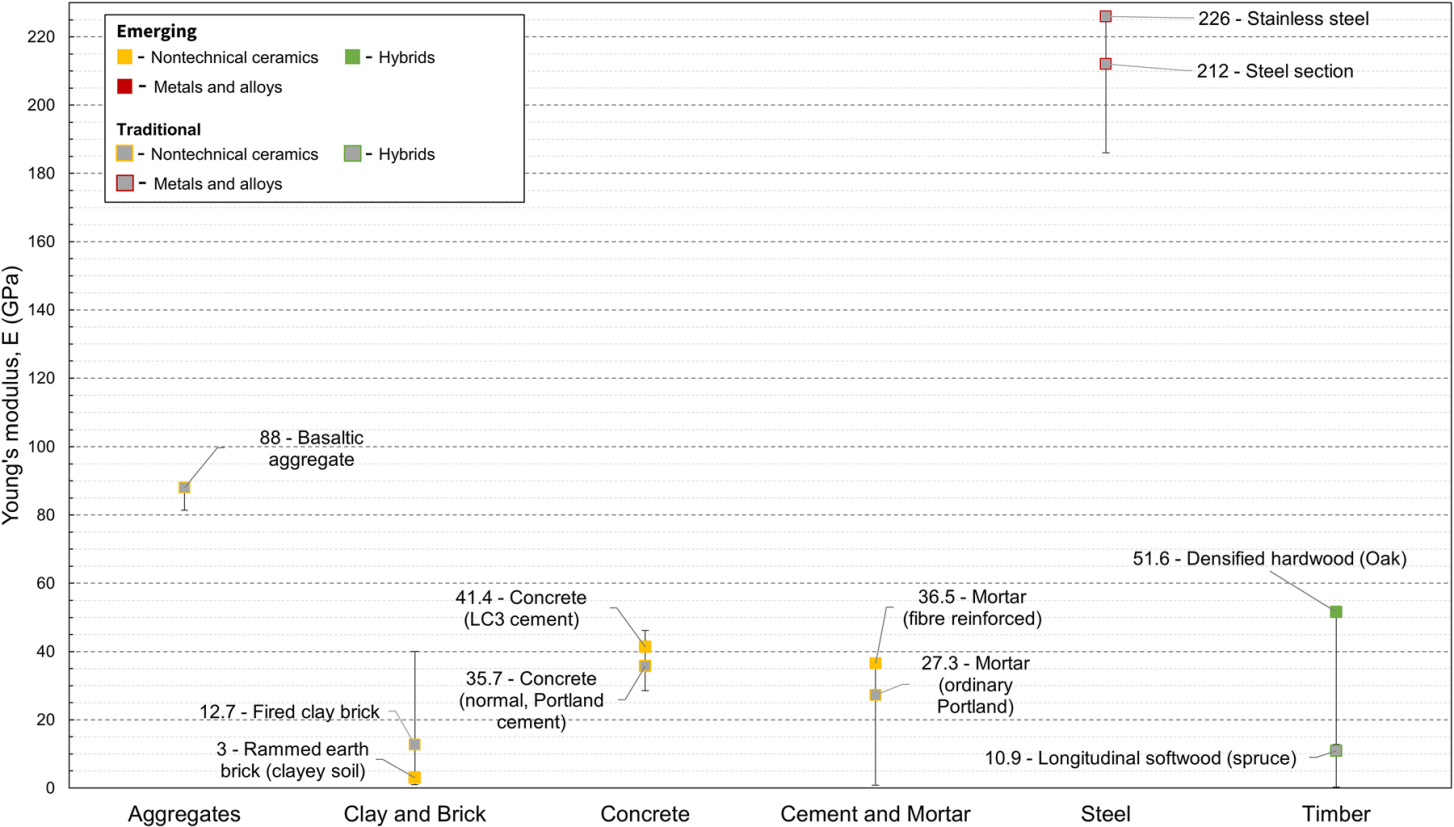
Highlighted Young’s modulus datapoints for six key material families showing emerging and traditional materials, where available. Differing production method are presented.

Figure 1 presents Young’s modulus (E) plotted against density (ρ). This combination of properties guides the selection of stiff and lightweight components through use of the material index lines (bottom right), which is covered in the section titled *Materials Selection Case Study*. Figure 2 highlights the range of Young’s modulus values in the dataset per material family and the variability within it.

Emerging concrete materials for which Young’s modulus property data were collected include limestone calcined clay concrete (LC3) (34–41.4 GPa) and self-compacting Portland cement-free concretes utilising ground granulated blast furnace slag (40.6 GPa)^18^. Other emerging concrete materials include compressed rubber (chipped tire) concrete which exhibited Young’s modulus values between 31.6 and 40 GPa for specimens with 10–15% rubber replacement^19^. A large range appears for fibre reinforced cement paste as this material group contained entries ranging from ordinary Portland cement substituted with carbon nanofibers (CNF) (38.1 GPa) ^20^ to wastepaper pulp fibre cement paste (0.7–1.5 GPa)^21^. Property data for other emerging cement-based materials, such as alkali-activated materials (AAMs), ultra-high-performance concrete and magnesium-based cement paste, were scarcer. Several high-strength stainless steels were included in the dataset. These include cold-formed ferritic and hot-rolled duplex with Young’s moduli ranging from 205.7 to 226 GPa. Emerging hybrid materials include cross-laminated timber (CLT) (4.74–16.7 GPa), densified wood (19.9–32.9 GPa), dowel laminated timber (10 GPa), structural composite timber (11.6–22.3 GPa), and bamboo (glue laminated, glulam) (7.4–13.3 GPa).

The mechanical properties of many of these emerging hybrid materials mentioned above match or exceed those of glulam timber (10.7–12.4 GPa) and should therefore be given greater consideration during the material selection process. The emerging concrete materials have similar properties to conventional concrete materials such as concrete (containing ordinary Portland cement, OPC) (28.5–46 GPa) and high-volume fly ash concrete (30.5–46 GPa). The highest values from the material property dataset are attributed to a CEM I cement concrete with basaltic aggregates (30–46 GPa)^22^. High strength steels generally exhibit greater Young’s moduli compared to low carbon steels (186–212.5 GPa). Low carbon steel refers to the carbon content of steel (~0.4%).

### Compressive strength, f_c_

**Fig. 3:**
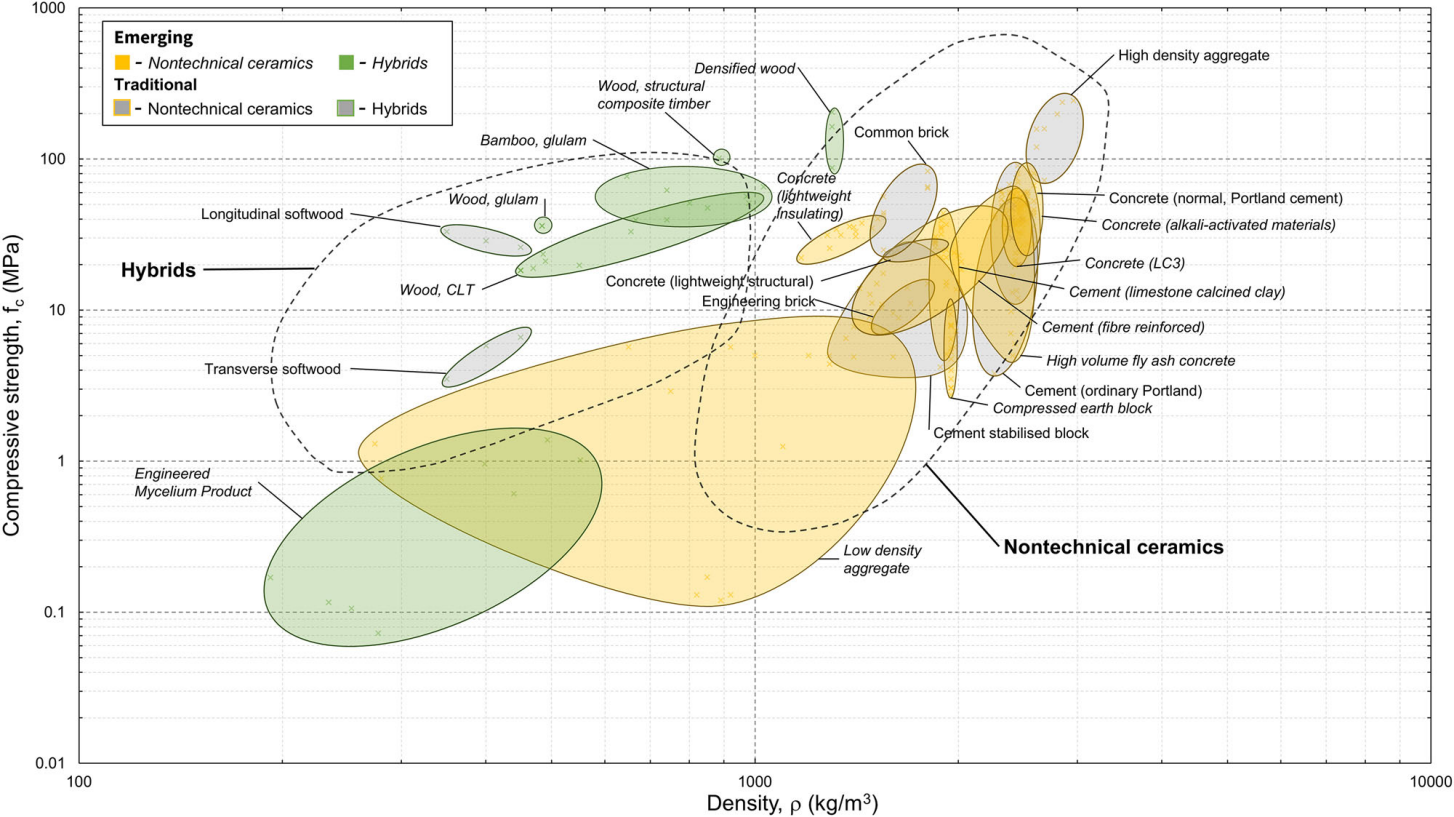
Material property charts for compressive strength (f_c_) with respect to density (ρ). The labelled ovals enclose material data collected for this study, with all datapoints plotted at 50% transparency. Ovals filled in grey enclose material property data for traditional materials, and ovals filled with colour (yellow, green) enclose material property data for emerging materials. Like Fig. 2, the dashed lines encompass material properties for conventional materials from a given material family from EduPack. Ovals plotted outside (or partially outside) of their respective material family extend the current materials property space. The definition of strength varies by material family; metals and polymers – yield strength (σ_y_), ceramics and glasses – modulus of rupture (MOR), hybrids – tensile failure strength (σ_t_).

**Fig. 4:**
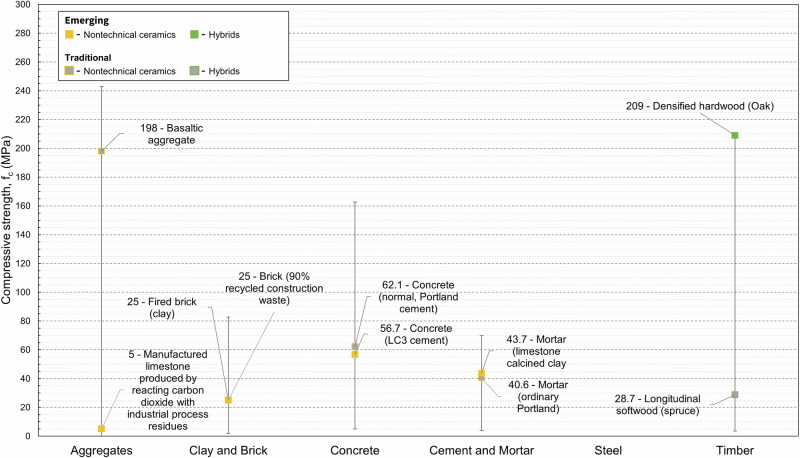
Highlighted compressive strength datapoints for five key material families showing emerging and traditional materials, where available. Data for differing production methods are highlighted. No compressive strength data was collected for steel.

Compressive strength is critical in construction. Structural integrity must be guaranteed as failure can often be catastrophic. Compressive strength is the second most recorded property in the dataset (208 datapoints). Figure 3 plots the compressive strength (f_c_) data against density (ρ) and Fig. 4 presents highlighted compressive strength datapoints. There are several materials with unknown densities which could not be plotted but are discussed in the text below, given their significance.

Due to the prevalence of concrete, cement paste, and mortar within the dataset, these materials account for almost two-thirds of the compressive strength data points collected. Typically, unconfined compressive strength at 28 days of curing is reported for these materials. Emerging cement pastes from the material property dataset include fibre reinforced cement paste (6.60–43.1 MPa), cement paste (limestone calcined clay, LC3) (14.5–43.7 MPa), and magnesium-based cement paste (22–69.8 MPa). The high strength values achieved for magnesium-based cements were made possible by microwave curing a magnesium phosphate hydrate cement paste^23^. Biocements are materials which make use of bacteria that bind together carbon and calcium to produce a biologically formed limestone. Initial results indicate that these materials, when compared to OPC, exhibit relatively high strength. One tile comprising 85% granite from recycled sources and 15% biologically grown limestone (calcium carbonate) exhibited compressive strength ranging from 27.6 to 41.4 MPa^24^. Further testing is needed to verify this manufacturers’ claim. Emerging concrete materials include AAM concrete (27.3–80.6 MPa) and LC3 concrete (20–60 MPa). Other specific examples include concretes utilising aggregate derived from i) granulated metal slag (60–68 MPa)^20^, ii) cement kiln bypass dust (28–35 MPa)^25^, and iii) post-consumer plastic waste (28.1–35.2 MPa)^26^. Furthermore, permeable (clog resistant) pavements (based on OPC) have been developed, that use a system of plastic tubes of varying diameter (3–6 mm), held in place using steel mesh, and facilitate water drainage (19–59 MPa) for porosities between 2–30%^27^. The compressive strengths of low-density aggregates range from 0.12 to 7.75 MPa for aggregates manufactured from incinerator bottom ash, sewage sludge ash, biomass ash, waste glass, clay and quarry fines or produced through carbonation of waste streams, e.g., cement kiln dust or thermal residue^28,25^. Compressed earth blocks exhibited low compressive strengths in the range 2–10.5 MPa. However, cement-stabilised earth building products showed greater strength (9.38–13.7 MPa). Narayanaswamy et al. (2020)^29^ demonstrated that compressive strengths of cement stabilised blocks increased when AAMs were used (12 M NaOH + 15% fly ash), and that the addition of construction demolition waste had no significant negative effect on the mechanical performance. A significant amount of data was collected for CLT (18.4–56.7 MPa). Its compressive strength increases with increasing wood density. Other hybrid materials include densified wood (87.6–163.6 MPa) and bamboo (glulam) (39.5–77 MPa). The material property dataset contained few entries for ordinary Portland cement mortar, and they exhibited a wide range of compressive strengths (3.8–40.6 MPa). This is due to the small sample size for this study (two datapoints) and the variability of constituents and their proportions, e.g., sand and water. A much larger amount of compressive strength data was collected for concrete. The compressive strengths of emerging concrete materials are within the property range of conventional concrete materials. Low-density aggregates understandably have much reduced strength compared to high-density aggregates (e.g., granite, limestone and basalt) (120–160 MPa). Compressed and unfired blocks generally exhibited compressive strength at the bottom end of the range of conventional alternatives such as common and engineered bricks. Conventional wood materials such a longitudinal softwood and glulam exhibited compressive strengths ranging from 28.7 to 36 MPa. This is significantly less than the values recorded for emerging engineered wood products (see above).

### Density

**Fig. 5:**
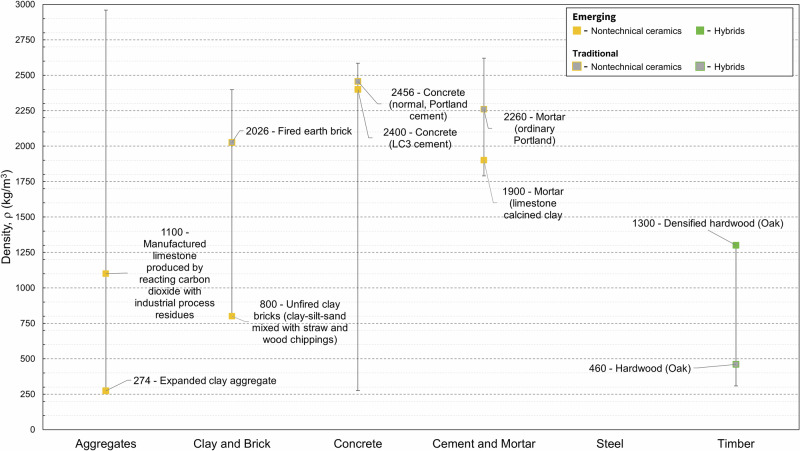
Highlighted density datapoints for five key material families showing emerging and traditional materials, where available. Data for differing production methods are highlighted. Steel has a narrow density range of 7850–7910 kg m^–3^ that lies outside the range of this plot.

Emerging lightweight aggregate densities range from 650 to 1600 kg m^–3^ (Fig. 5). This range includes lightweight aggregates derived from ashes (incinerator bottom ash, biomass ash), waste glass, quarry fines, and from carbonation of stabilised thermal residue, such as industrial process residues^28^. Commonly used lightweight aggregates, such as expanded clay aggregate have lower density (260–610 kg m^–3^) than their emerging counterparts. Other aggregates include secondary aggregate (from granulated blast furnace slag, copper slag, waste plastic) (2380–2387 kg m^–3^) technologies.

Emerging cement pastes, mortars, and cement-based construction materials include fibre reinforced cement (1430–2261 kg m^–3^). The large range is due to cement paste composites on the lower end with significant wastepaper pulp fibre content and conventional cements and carbon nanofibre reinforced mortar on the upper end. Other materials included LC3 paste and mortars (1850–1920 kg m^–3^); cement stabilised earth blocks (1,880–2010 kg m^–3^); and compressed earth blocks (800–2397 kg m^–3^), with the large range explained by lightly compacted (low end) and hyper compacted (high end) unfired earth bricks^30^; and LC3 concrete (2367–2456 kg m^–3^), and alkali-activated materials (2468–2585 kg m^–3^). The large density range for fibre reinforced cement paste is due to the inclusion of low-density waste bio-fibre cement-based composites reinforced with nano-SiO_2_ particles^20^. There was a lack of density data collected for common cement pastes and mortars, given that these materials are generally well characterised in the literature. Densities of concrete materials are similar within the same sub-class. E.g., emerging general purpose concrete materials have similar density to conventional PC based concrete, which are significantly different from light weight (structural, 1570–1840 kg m^–3^; insulating, 275–1520 kg m^–3^) and high-performance concretes (2531 kg m^–3^). The large density range for light weight insulating concrete is due to the inclusion of hemp and miscanthus based concrete blocks with a hydrated lime binder in the dataset, which have significantly lower values than other types of insulating concrete, that have densities of 1170 to 1520 kg m^–3^^31^. Densities for engineered wood products were collected for cross laminated timber (CLT) (437–972 kg m^–3^), densified wood (403–1300 kg m^–3^), dowel (mechanically) laminated timber (550–590 kg m^–3^), bamboo (glulam) (620–1030 kg m^–3^), and structural composite timber (885 kg m^–3^). The large density range for CLT is due to the inclusion of high-density coconut wood in laminated timber samples, which led to significantly higher values than the other types of CLT, which had densities of 437–550 kg m^–3^^32^. Densities of engineered wood products greatly depend on the species of timber used. In general, engineered wood products have a greater density than the woods from which they are derived due to the use of adhesives and densification during their manufacturing

### EC and energy

**Fig. 6:**
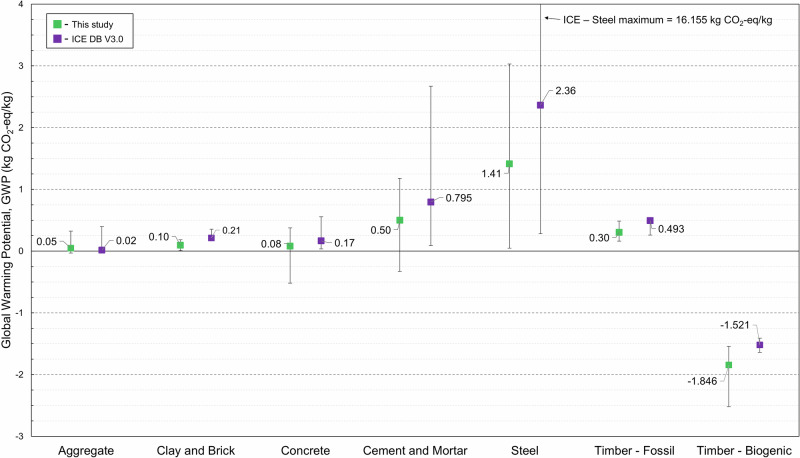
Embodied carbon (EC) for six major materials groups; aggregates (*n* = 5), clay and brick (*n* = 4), concrete (*n* = 31), cement and mortar (n12), steel (23), timber (*n* = 8), normalised to a functional unit of 1 kg. EC data is all product stage (A1–A3) in line with BS EN 15804^46^. Groups relate to those within the ICE database V3.0^17^. Labelled values are averages; extended bars show minimum and maximum global warming potential (GWP) within each dataset.

EC data from the materials property dataset is presented in Fig. 6 using production stage environmental impact data from BS EN 15804 and ISO 14040 (BSI, 2019; ISO, 2020a), normalised to a functional unit of 1 kg. For comparison, literature data from the ICE database V3.0, where such data were available for similar material groups, are included in Fig. 6^17^. There is a close correlation between data points in the dataset developed here and those in the ICE database V3.0, validating the former. While construction products have environmental impacts throughout their lifecycle (as discussed in the section titled ‘*Other Life Cycle Considerations*’), this information is often semi-quantitative given it is common for only the product stage (A1–A3) to be assessed and reported. Challenges also exist regarding the transparency and reliability of background lifecycle inventory data in general, leading to uncertainties^33,34^.

**Fig. 7:**
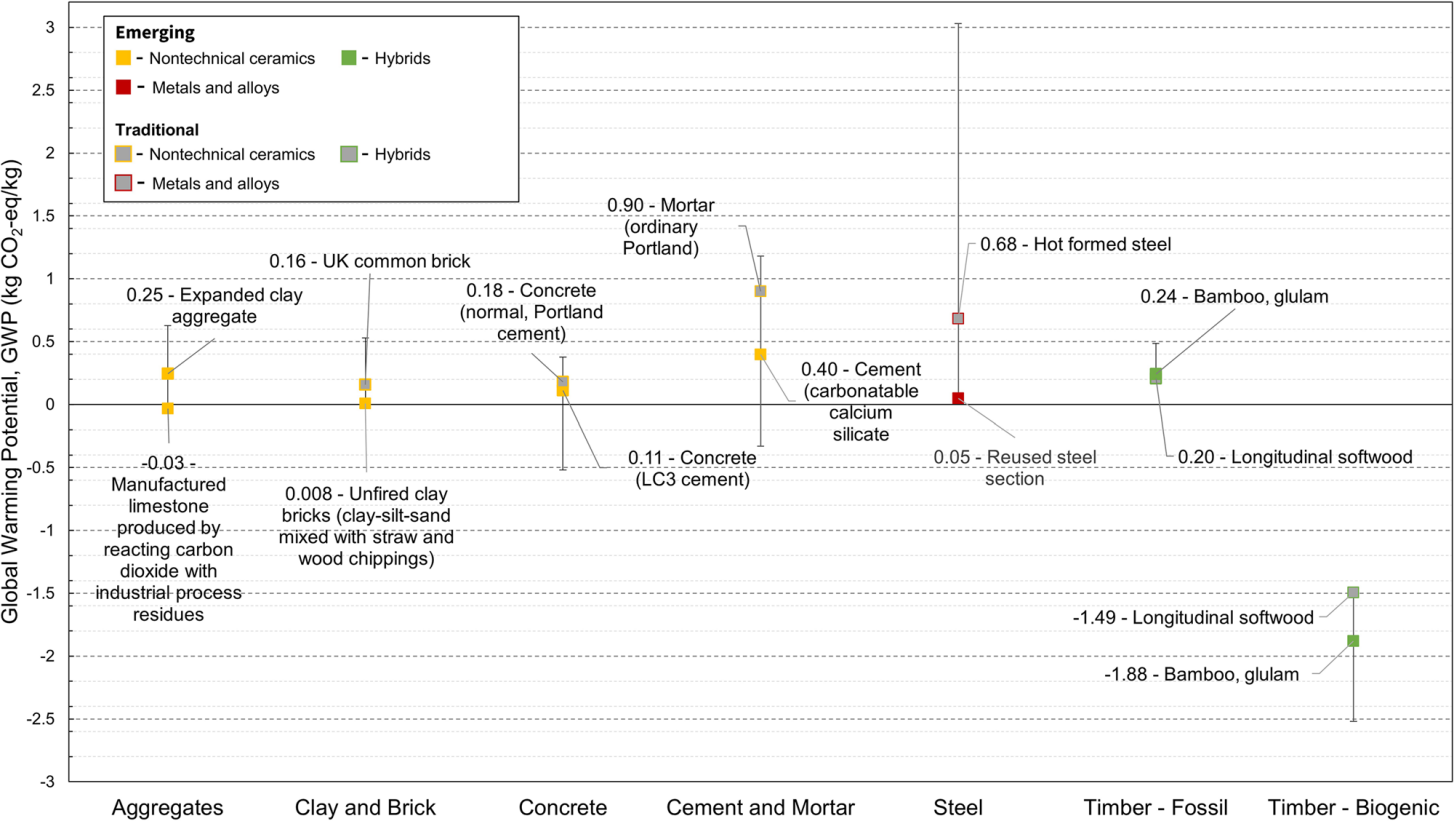
Highlighted global warming potential datapoints for six key material families showing emerging and traditional materials, where available. Data for differing production methods are highlighted.

**Fig. 8:**
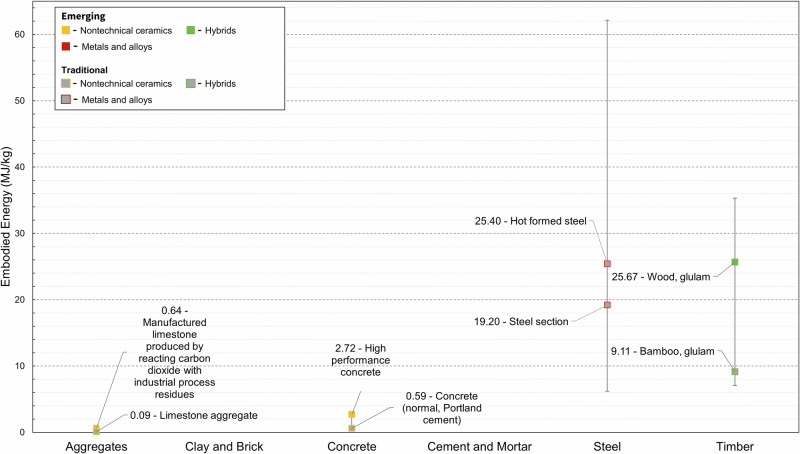
Highlighted embodied energy datapoints for four key material families showing emerging and traditional materials, where available. Data for differing production methods are highlighted. Embodied energy data were unavailable for ‘Clay and Brick’ and ‘Cement and Mortar’.

Out of 409 material candidates included in the dataset only 113 (27.6%) had embodied carbon data available. Embodied energy data were only available for 37 (9%) entries. Lack of this data is a huge challenge, especially for assessing the environmental sustainability of emerging materials. Fig. 7–8 show comparisons between the embodied carbon and energy values in the dataset for emerging and traditional materials.

The average GWP for aggregates, clay and brick are very close to those reported in the ICE database V3.0, which is expected since these are well-established industries which publish sufficient data^17^. A compressed brick with 90% construction demolition waste content was reported to have a GWP of 0.07 kg CO_2_-eq./kg^35^. EC values for aggregates derived from post-consumer plastic waste were collected (0.324 kg CO_2_-eq./kg)^26^. The material responsible for the negative aggregate GWP value of –0.046 kg CO_2_-eq./kg is a low-density aggregate created via accelerated carbonation of thermal residue^28^. Carbon capture, utilisation, and storage is being deployed on naturally reactive materials such as incinerator bottom ash, sewage sludge, and waste drill cuttings. Residues are exposed to high concentrations of carbon dioxide in the presence of moisture. Uncarbonated CaO in the residues such as in portlandite (Ca(OH)_2_) combine with dissolved CO_2_, yielding carbonate e.g., CaCO_3_. For low density aggregates the CO_2_ uptake by weight of aggregate ranges from 4–8.5%^36^. Alternative cement binders are reported by Miller and Myers (2021)^37^, of which the lowest impact is magnesium oxide cement binder derived from Mg_2_SiO_4_ (–0.33 kg CO_2_-eq./kg) with CO_2_ curing, which was the lowest cement binder GWP datapoint collected here. Alternate concretes included in this study show a small decrease in the average EC, with the minimum achievable value being much lower: EC reductions up to 75% are possible with AAM concrete due to their reduced Portland clinker content through its replacement by fly ash, calcined clay, etc., however there is a lack of EPD data on these materials—especially in the UK^38,39^. There is also a reduction in the EC of concrete through the inclusion of miscanthus and other natural fibres (e.g., hemp). Hydrated lime/natural hydraulic lime binders to produce low-strength insulating concrete blocks are reported to have EC values ranging from –0.52 to –0.25 kg CO_2_-eq./kg (biogenic carbon of miscanthus canes included)^40,41^. Finally, magnesium hydroxy-carbonate hydrate based concrete is reported to have a low EC value, since it can absorb substantial quantities of CO_2_ (up to 30 wt.% CO_2_)^42^. For steel production the EC values collected in this study contain datapoints from anticipatory (ex-ante) LCAs where the inclusion of a renewable energy source has a significant impact^43^. This explains the low average (1.41 kg CO_2_-eq./kg) EC value for the developed dataset compared to the ICE dataset (2.36 kg CO_2_-eq./kg) (Hammond and Jones, 2019). Reuse of steel has by far the lowest impact. Steel reuse involves collecting, cleaning, and testing used sections, allowing them to be reused structurally with very low EC emissions (0.047 kg CO_2_-eq./kg)^44^. For timber, all entries have separate biogenic and fossil carbon. The fossil EC for timber in this study ranges from (0.16 to 0.49 kg CO_2_-eq./kg) which is similar to the range presented in the ICE database (0.26 to 0.51 kg CO_2_-eq./kg)^17^. There is a lack of information in the literature on the EC of densified wood and for this reason it has not been included in the case studies here. One study was found that reported that the densification process did not make a large contribution to the overall emissions compared to drying and transport, and suggested that these materials would be particularly suitable for high load applications^45^.

### Other life cycle considerations

Several factors can affect the functional and environmental properties of materials and data included in our database. For example, the dataset has inherent uncertainties due to representativeness of datapoints across geographies, material sourcing, transportation, and processing approaches, energy grid and process emissions, and certification approaches used for underlying materials. However, such limitations are common across life cycle databases as noted by Guo et al. (2025) and Xu et al. (2025)^33,34^.

To highlight that our dataset captures such factors, we showed the effects of manufacturing methods on the material properties of six material families: Young’s modulus in Fig. 1, compressive strength in Fig. 3, density in Fig. 5, and EC and energy in Figs. 7 and 8. We find that such variability influences the environmental performance of a specific material candidate but within the range for the broader material family. Data on several other material properties were collected, and the results are included in Supplementary Dataset 1. Properties include shear strength, shear modulus, health performance, and fire rating.

Additionally, semi-quantitative information not captured within BS EN 15804 product stages A1-A3^46^, e.g., about maintenance, durability, service-life, end-of-life, reuse, and recycling, is important in materials selection but often not disclosed in construction material EPDs^46^. Nevertheless, deleterious processes affecting construction materials are important since they inform maintenance, repair schedules, and, in turn, service life. Several emerging materials within the dataset lack EPD data, but their potential in lowering EC has been proven. Therefore, we consider these factors here for several major material classes (nontechnical ceramics, hybrids and natural materials, and metals and alloys), which we now discuss.

For nontechnical ceramics such as concrete, carbonation is important since it can affect their EC values. These materials can be carbonated during curing ‘forced’ or during service life ‘natural’. Natural carbonation is a process that occurs throughout the life cycles of cementitious materials. CO_2_ is absorbed by these materials during storage, use, and at end-of-life. Typically, carbonation of concrete during use and at the end of life is not included in the scope of LCA studies for concrete. For example, the EC of miscanthus concrete blocks recorded in Supplementary Dataset 1 is 42% lower when carbonation of the block over a 100-year life cycle is considered^41^. However, given the system boundary for production that is often used to estimate EC (i.e., A1–A3), carbonation during use and the end-of-life phase is omitted from the scope here.

Hybrid and natural materials, such as engineered wood products derived from timber, can be associated with CO_2_ sequestration during the growth phase prior to felling, or after felling and during tree regrowth. This is referred to as storage of biogenic carbon, and in the former case is represented as a negative GWP value during the A1 module of an LCA (assuming timber is sourced from sustainably managed forests)^46^. If timber is reused at end-of-life, CO_2_ will remain stored, though this reuse may affect primary timber demand. If disposal or incineration occurs, then carbon is released to the atmosphere and represented as an emission. This is an important but complex issue that has received significant academic and industry attention, as explored in recent reports and studies on methodical challenges, policy frameworks, and carbon flow modelling^47,48^. For example, changes to BS EN 15804 + A2 made in July 2022 split the climate change impact category into four (total, fossil, biogenic, and land use and land change)^49^, and made reporting of modules A1–A3, C1–C4, and D, and of biogenic carbon for materials containing more than 5% biogenic carbon mandatory^46^. This issue of how best to account for biogenic carbon relates to the complexities of forestry practices and service lifetime estimations.

Moisture exposure is a critical factor in selecting engineered wood products for structural components such as beams and columns, since moisture uptake can happen during construction, use, and at end-of-life. Relatively high moisture levels, typically >18-20% wood moisture content, lead to dimensional swelling and a potential decrease in effective density due to volumetric expansion outpacing mass gain from water absorption, which in turn reduces key mechanical properties like modulus of elasticity, bending strength, and compressive strength^50–52^. This is particularly relevant for beams, where reduced bending strength compromises load-bearing capacity. Addressing these moisture-density interactions in material property databases is complex but essential for accurate structural design and selection, and could be prioritised in future work.

Regarding metals and alloys, several factors may affect their EC values, which introduce uncertainty into the data collected. These include considerations around steel reuse, such as availability, processing, transportation, and certification requirements. Increasing scrap steel collection for recycling and reuse, and the use of electric arc furnaces (EAFs), present immediate opportunities to reduce the EC of steel. Manufacturing methods can also have implications, for example, additive manufacturing of constructional steels. Material properties of printed components have been verified^53–55^, and it has been shown that wire arc additive-manufactured steel beams have a lower carbon footprint when compared to steel I-beams under a range of conditions, which notably depend on mass reduction^56^. Other issues include corrosion and cracking, which may require additional maintenance and replacements. These are often not considered within a typical material performance assessment.

### Materials selection case studies

The materials property database developed here was applied to screen candidates using Ashby’s indices (Supplementary Information) before detailed evaluation in two case studies. Initial screening via charts (e.g., Fig. 1) highlighted high-E/ρ materials like steel for stiffness, but integrating EC shifted preferences towards low-density, low-EC options like timber and reused steel (Table 3). Case studies quantified functional equivalence, with calculations using Eqs. 1–4 and shown in Supplementary Dataset 1, σ_design_ = σ_c_/1.5 (γ_m_ = 1.5 uniform for simplification), and uplifts (0 for concrete, 0.1 for timber, 0.15 for steel). Variants analysed include both traditional and emerging materials from the dataset: steel, wood (glulam & CLT), bamboo (glulam), and concrete (LC3, Portland, and high volume fly ash cement). Ranks reflect ascending total EC.

A second sustainable material index incorporating EC is also presented. Representative EC data for structural densified wood were not available in the literature.

**Table 3:** Results from ranking several candidate materials for a light (mass minimised) simply supported beam and column, where performance is governed by the material index E^1/2^/ρ

Material	Traditional / Emerging	Material index (E^1/2^/ρ)	Material index rank	Sustainable material index (E^1/2^/ρ*EC)	Sustainable material index rank
Steel	Traditional	0.058	8=	0.036	8
Reused steel	Emerging	0.058	8=	1.246	1
Densified wood	Emerging	0.132	4	n/a	n/a
Bamboo, glulam	Emerging	0.143	3	0.593	6
Wood, CLT	Emerging	0.207	2	0.627	4
Wood, glulam	Emerging	0.214	1	0.614	5
Concrete (LC3)	Emerging	0.080	6	0.730	2
Concrete (normal, Portland cement)	Traditional	0.074	7	0.528	7
High volume fly ash concrete	Emerging	0.081	5	0.672	3

Results for the floor-beam case study are shown in Table 4. All designs were strength-governed, as low flexural strengths (MOR) dominated over deflection for the 9 m span. Emerging variants like reused steel and bamboo glulam yielded the lowest EC total (160–177 kg CO_2_-eq.), with masses (668–2987 kg), leveraging low density/EC. Reused steel ranked first (160 kg CO_2_-eq.), a 97% reduction vs. traditional steel (5497 kg CO_2_-eq.) due to ultra-low EC (0.047 kg CO_2_-eq./kg). Timber CLT/glulam followed (238–268 kg CO_2_-eq.). Concrete required large sections (h_req_ = 1.55–1.64 m) from approximated MOR (per ACI 318), resulting in high masses (10,249–10,637 kg) and EC (1154–1438 kg CO_2_-eq.), though emerging LC3 and fly ash mix designs outperform normal Portland cement concrete.

**Table 4:** Beam case study results. Embodied carbon total includes 10% uplift for timber and 15% uplift for steel

Material	Traditional / Emerging	Required beam height, h_req_ (m)	Required cross-sectional area (m^2^)	Mass (kg)	Embodied carbon (kg CO_2_-eq.)	Rank
Steel	Traditional	0.140	0.042	2987	5497	8
Reused steel	Emerging	0.140	0.042	2987	160	1
Bamboo, glulam	Emerging	0.334	0.100	668	177	2
Wood, CLT	Emerging	0.563	0.169	739	268	4
Wood, glulam	Emerging	0.474	0.142	619	238	3
Concrete (LC3)	Emerging	1.619	0.486	10,494	1154	5
Concrete (normal, Portland cement)	Traditional	1.550	0.465	10,249	1438	7
High volume fly ash concrete	Traditional	1.642	0.492	10,637	1276	6

Results for the column case study are shown in Table 5. Designs were buckling-governed for high-*E* materials (e.g., steel, bamboo/glulam) and strength-governed for others, reflecting compressive demands. Reused steel minimised total EC (77 kg CO_2_-eq.) with compact sections (*A*_*req*_ = 0.052 m^2^), outperforming traditional steel (2637 kg CO_2_-eq.) by 97%. Timber variants followed (149–278 kg CO_2_-eq.), with low masses (388–605 kg) from density advantages; wood glulam ranked second. Concrete achieved moderate EC (174–201 kg CO_2_-eq.) via strength governance, with emerging LC3 concrete being the lowest of the concrete materials considered here.

**Table 5:** Column case study results. Column diameter assumed circular section. Embodied carbon total includes 10% uplift for timber and 15% uplift for steel

Material	Traditional / Emerging	Required column cross-sectional, A_req_ (m^2^)	Required column diameter (m)	Mass (kg)	Embodied carbon (kg CO_2_-eq.)	Rank
Steel	Traditional	0.052	0.257	1433	2637	8
Reused steel	Emerging	0.052	0.257	1433	77	1
Bamboo, glulam	Emerging	0.225	0.535	584	154	3
Wood, CLT	Emerging	0.355	0.673	605	219	7
Wood, glulam	Emerging	0.229	0.540	388	149	2
Concrete (LC3)	Emerging	0.189	0.490	1585	174	4
Concrete (normal, Portland cement)	Traditional	0.158	0.449	1357	190	5
High volume fly ash concrete	Traditional	0.199	0.504	1673	201	6

## Discussion

This study collated and reported material property data for emerging structural materials for construction, focusing on those that have lower EC and exhibit equivalent or improved functional performance. This material property database highlighted the promising nature of emerging hybrid and nontechnical ceramic materials for structural applications. For concrete, both LC3 and magnesium-based cements showed comparable Young’s modulus and compressive strengths to conventional ordinary Portland cement concrete. Emerging engineered wood products, such as glulam, CLT, dowell laminated timber, densified wood, structural composite timber, and bamboo (glulam) outperform conventional softwood/hardwood and glulam based on their Young’s moduli and compressive strength.

The results reported here demonstrate the value of the database in identifying low-EC materials for construction, extending Ashby’s indices by quantifying functional EC and mass. Emerging materials had the best performance across the case studies: reused steel/bamboo glulam has the lowest EC for beams (160–177 kg CO_2_-eq.), and reused steel/wood glulam for columns (77–149 kg CO_2_-eq.), achieving 92–97% reductions vs traditional counterparts. The low density of timber (484-1300 kg/m^3^) drives its resource efficiency (mass 388–739 kg, 60–95% below concrete/steel). Emerging options like LC3 concrete (174 kg CO_2_-eq.) and bamboo glulam (154 kg CO_2_-eq.) offer 8 and 19% EC savings compared to normal Portland cement in the column case study.

The low EC of reused steel underscores a benefit of circularity in the steel life cycle, although this is reduced somewhat by EC uplifts for fire protection. The availability of reused steel, and its processing and certification, may, however, pose challenges in adoption at scale.

The high resource efficiency of timber, with its reduced extraction and transportation of mass compared to concrete, supports industry guidance (Institution of Structural Engineers, The Steel Construction Institute) for hybrid systems. Factors to consider in interpreting the results for timber here include the use of idealised sections (rectangular/circular) rather than I-beams and CLT in the case studies, which could cut mass utilised in sections by 20–45%; the MOR approximation for concrete (ACI 318); no vibration or fire details, which are important in real designs; and the simplistic EC accounting used (e.g., which does not consider the end-of-life stage); and the 9 m span, which suits timber/steel but penalises concrete.

Overall, the study demonstrates the database's applicability in assisting low-EC selections in multi-story buildings, favouring emerging timber and reused steel over other common structural materials. However, several challenges remain for broader emerging materials data. First, developing material databases requires extensive resources and expertise. The datapoints are highly disparate and bringing them together remains a complex challenge. Academic and industry collaboration is key to delivering such datasets, since emerging materials are often in the early stages of commercialisation, and often updates to these should be jointly coordinated. Secondly, the availability of environmental performance data for emerging materials is still uncommon as an industry practice, validated by the fact that only 27% of the material candidates in our database have EC values, and 9% have embodied energy values. Further complications exist in differentiating biogenic and fossil emissions in the environmental data, which, if combined, can be misleading. Thirdly, we have shown that the selection of an appropriate functional unit that is not mass-based (e.g., 1 kg) is key for fairly comparing environmental performance. It is thus crucial that designers and developers properly develop comparisons on component bases to draw conclusions on material choices, which would require close collaboration between stakeholders, including material providers, designers, developers, and owners.

Finally, the consideration of emerging material properties and their environmental impacts needs to be better recognised in decarbonisation policies for the built environment. Testing and certification requirements can often be a limiting factor for several industry members. There is a need for better support to facilitate material testing and certifications for emerging materials that deliver better performance at lower environmental impacts. Our dataset demonstrates that key material property extensions can be delivered by emerging materials; future work can focus on discovering, developing, and recording the properties of more emerging materials, especially those that have properties distinct from traditional materials.

Therefore, this paper shows the benefits of collecting material property and EC data for emerging structural materials, and likely also in other application domains. The database developed in this study can help material innovators, designers, and developers to make effective material choices, scale the adoption of emerging materials and advance global efforts to deliver a low-carbon built environment.

## Methods

### Data

Data on existing and novel construction materials were collected and analysed following the research methodology presented in Fig. 1. Material property and environmental impact data on construction materials were collected through a literature review. Information was extracted from relevant papers and compiled to facilitate comparison. The dataset was further supplemented with product manufacturer test data and third-party-verified information, including environmental product declarations gathered through a survey of 60 companies. Data are generally presented here ‘as found’ or with minimal unit conversions. For example, volume data, e.g., m^3^, were converted to mass (kg) using density data. The material property and environmental impact data collected are shown in Supplementary Dataset 1.

**Fig. 9:**
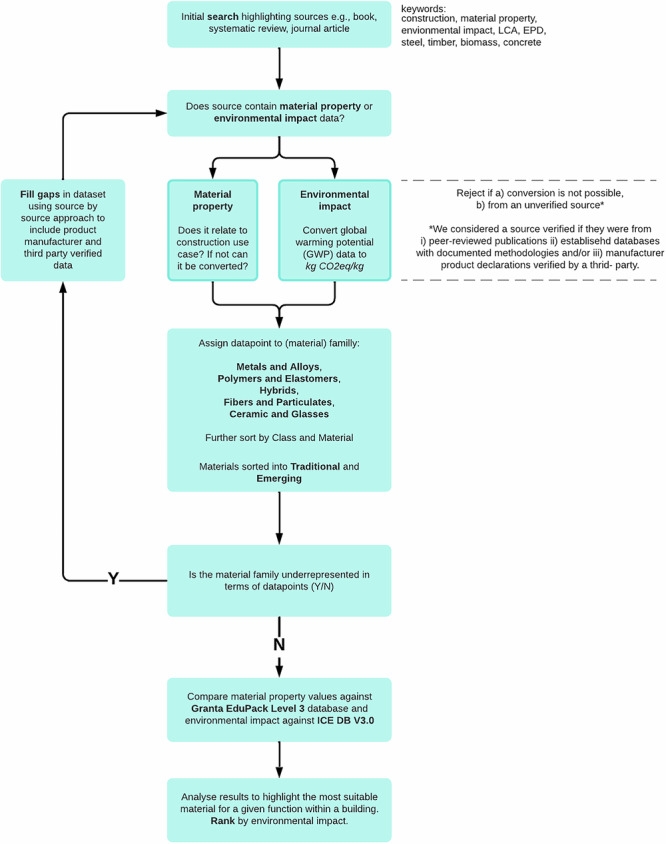
Flow chart showing how performance and environmental impact data on structural materials were collected in this study. Class is defined as the level below material family, for example the two classes of alloys are ‘ferrous alloys’ and ‘non-ferrous alloys’. Material refers to groups of data points that are chemically and physically equivalent at the relevant class. Materials are sorted into Traditional and Emerging; this categorisation is provided in Supplementary Dataset 1.

Specifically, the data come from 155 unique sources that were published between years 2005-2022. In our search we used the keywords (construction, material property, environmental impact, LCA, EPD, steel, timber, biomass, concrete) with inclusion and exclusion criteria noted in Fig. 9 that relates to the robustness of the sources. All sources are available in Supplementary Dataset 1. The data we reconciled was primarily based on material properties that were used to develop the database.

Materials that deliver structural functionality were prioritised. Concrete is the most numerous material within the dataset with 117 individual entries. All mechanical properties of concrete and cement paste and mortar were recorded at 28 days of curing. This was chosen as most of the strength development in concrete typically occurs by 28 days of curing and material properties at this curing time are widely reported across different sources. Non-structural, façade materials, and internal finishes were not considered due to their lesser contributions towards embodied carbon. Additionally, glass was not included, given the homogeneity in its properties and that novelty in this material class generally arises from coatings and interlayers rather than from the bulk glass. Several materials in the database have low technology readiness levels (TRLs). For materials at these stages of development, there is a lack of third-party verified material property and environmental impact data. To obtain this data, we contacted 60 companies, from which there were 17 responses. There were 15 positive responses, 9 of which provided data that are used in this study. Negative responses cited privacy issues around data sharing, or the organisation had not developed their product to a level where they were happy to share material property information.

Information was collected on 21 different material properties (summarised in Table 2), focusing on mechanical property data, e.g., modulus of elasticity (*E*), compressive strength (*f*_*c*_), tensile failure strength (*σ*_*t*_), yield strength (*σ*_*y*_), and flexural strength (MOR), since these are especially important for structural applications. Some data on properties such as moisture content, water absorption, and pH were also collected. Environmental data focused on the cradle-to-gate 100-year global warming potential (GWP100) for a functional unit of 1 kg, specifically encompassing embodied carbon from lifecycle stages A1-A3 (raw material extraction, transport, and manufacturing – cradle-to-gate) as defined under the ISO 14040 standard. Where available biogenic and fossil carbon were collected for all bio-based materials (*n* = 8) and other materials (*n* = 11), however we found that combined (fossil and biogenic) impact values are prevalent across the material categories including that for bio-based materials. CO_2_ uptake (reported in mass%), and semi-quantitative information about maintenance, durability, and service life were also collected. CO_2_ uptake refers to the carbonation process where CO_2_ reacts with calcium-based compounds, such as concrete, to form calcium carbonate and it is defined as percentage of carbon dioxide absorbed by a material through carbonation.

For construction products, material property data are presented in both technical data sheets and safety data sheets. Existing databases such as *Ansys® Granta EduPack v2022R2* contain extensive property data on a wide range of materials^16,57,58^. There is however a lack of LCA data associated with entries and hence alternative databases such as the ‘Inventory of Carbon and Energy’ (ICE) and ‘ecoinvent’ are widely used in the construction industry^17,59^. ICE is used by many researchers and organisations (e.g., ‘One Click LCA’) as the background EC database for Whole Building Lifecycle Assessments (WBLCA). It is therefore important to assess the materials it contains, given its influence on building-level EC calculations in practice. ICE database aggregates environmental data from EPDs as well as industry average material property data from the CIBSE guides^60^, but the variety of alternate materials is limited.

Two forms of environmental impact data were collected from i) LCA studies reported in journal papers, and ii) third-party verified construction product environmental product declarations (EPDs) consistent with BS EN 15804^46^. Different types of EPDs exist. These include industry or sector average, organisation average, and single organisation product specific (i.e., ‘*type III environmental product declarations*’). In the authors’ experience, the globally accepted standard within construction is BS EN 15804; however, in the United States, the use of ISO 21930 is also common^61^. Since the reliability and transparency of environmental impact data is important given the drivers to reduce EC, independent verification, in the form of third-party verification, is a differentiator for high quality construction product environmental impact data. Unverified EPDs and in-house sustainability assessments are typically of lower quality.

The functional unit (FU) of environmental impact data varied between sources; therefore, we converted all the data into a declared unit of 1 kg material. The system boundary for the environmental impact data is the product stage (A1-A3). This includes impacts from raw material extraction, processing, transport to the manufacturing site, and manufacturing.

*Ansys® Granta EduPack v2022R2* does contain environmental impact data; however, these data lack transparency regarding system boundary definition, making comparisons between materials challenging. For example, EduPack does not refer to life cycle stages and it is unclear whether carbon footprint data for materials within EduPack covers the same life cycle stages (with the same specific boundary definitions) compared to those found in BS EN 15804. Impact data relates to material production with no further detail regarding the production process. Data sources for EduPack include ecoinvent v2.2^62^ or ICE database v1.6a^63,64^.

Data were analysed here comparing collected material property data for specific material categories and not for specific materials, where possible, against the *Ansys® Granta EduPack v2022R2* ‘Level 3’ database^16^, whereas collected environmental impact data were compared to the ICE database V3.0 (Hammond and Jones, 2019) to highlight the novelty in emerging material properties that expand the existing coverage and/or data range. Within *Ansys® Granta EduPack v2022R2* there are several databases: Level 3 was chosen as it offered the greatest detail (>3900 materials) and is used in both university teaching and real design projects^16^.

### Materials selection using material indices and case studies

To demonstrate the applicability of the materials property database developed in this study, we initially applied Ashby’s material index methodology, as outlined in ref. ^65^. The material index is defined as a performance metric derived from the governing equations of a component’s function, constraints, and objective. This index is useful for initial screening of candidate materials that could deliver the required structural and/or functional performance. Material properties are raised to exponents determined by the scaling relationships in the performance equation and combined into a single metric. For example, in stiffness-limited designs under minimum mass objectives, the index often takes the form shown in Eq. 1 for beams or columns prioritising materials that provide high stiffness relative to density. This approach enables rapid screening of candidate materials (e.g., Ashby charts plotting *E* vs. *ρ* with materials index guidelines shown in Fig. 1).

1 $$M=\frac{{E}^{1/2}}{\rho }$$

Examples of common material indices derived in Ashby (2017)^65^ are provided in Supplementary Information, illustrating applications for structural components like ties, beams, and columns under various constraints (e.g., stiffness- or strength-limited designs at minimum mass. These metrics offer valuable insights into intrinsic material performance; however, they typically focus on mechanical efficiency and do not fully integrate environmental metrics like EC or practical provisions (e.g., fire protection, which adds mass and emissions via coatings). To address this limitation and provide a more comprehensive assessment incorporating functional equivalence, resource efficiency, and total EC (including uplifts for protections), two case studies were developed. We considered a beam fixed at both ends and a column under compression to analyse the benefits from the use of our emerging materials dataset. Simultaneously evaluating functional performance and environmental impact allows for the selection of materials with the lowest EC.

Both case studies ensured functional equivalence across materials (timber, concrete, steel variants) by designing to identical performance requirements per British standards (BS EN 1990, BS EN 1991-1-1). The primary objective was minimising total EC. Cradle-to-gate (A1-A3) data were used and biogenic carbon was reported separately from fossil carbon. We also considered protections applied to structural materials; steel typically has fire-proof paint and timber has varnish. Therefore, we uplifted the EC figures for timber by 10% and steel by 15% in line with similar case studies in the literature^66–68^ (an EC uplift of 0% was used for concrete).

For the floor–beam system, the functional unit was “one secondary beam supporting a 9 m span under ULS load = 33.76 kN/m and SLS load = 19.5 kN/m, with deflection ≤ L/360 = 25 mm, and 90 min fire resistance”. A rectangular cross-section (width b = 0.3 m) was assumed for normalisation. Calculations followed Ashby’s equations adapted for bending (design moment, Eq. 2; strength-limited height, Eq. 3):

2 $${M}_{{ed}}=\frac{{w}_{{ed}}\,{L}^{2}}{8}$$

3 $${h}_{{strength}}=\sqrt{\left(\frac{6\times {M}_{{ed}}}{b\times {\sigma }_{{design}}}\right)}$$

A limitation of this case study is the application of concrete in a simply supported beam. Concrete exhibits low flexural strength and the data have limited flexural strength data. For this reason, an approximation for flexural strength was taken from the compressive strength data collected using the ratio set by ACI 318 of 0.62√σ_c_. In reality concrete is combined with reinforcing steel bar which has high tensile strength, however this case studied considered single materials only.

For the column system, the case study used the functional unit “one axial column (length = 3.5 m, effective buckling = 3 m) resisting a force = 5 MN, 90 min fire resistance”. A circular cross-section was assumed for normalisation. Calculations included the buckling limited area (Eq. 4):

4 $${A}_{{buckling}}=2\times {L}_{e}\times \sqrt{\frac{\pi \times {N}_{{ed}}}{E}}$$

Limitations of the case study include its use of idealised sections, and further work could explore hybrid systems, e.g., steel reinforced concrete.

## Supplementary information

Supplementary Information

Supplementary Data 1

## Data Availability

Data collected and analysed in this paper are provided in Supplementary Dataset 1, which also includes information on the sources where the data were obtained.
