# Supplementary material for: Sustainable materials selection with emerging structural materials
Source: Npj Mater Sustain. 2026 Apr 10;4(1):13. doi: 10.1038/s44296-026-00099-7 (PMC13068519; doi:10.1038/s44296-026-00099-7)
Supplement: Supplementary file 1 — Supplementary Information [file 44296_2026_99_MOESM1_ESM.pdf]

Supplementary Information: Sustainable materials selection with emerging structural materials

Sam Burdett[1,2,a] , Mohit Arora[1,3,b], Rupert J. Myers[1,c,*]

[1] Department of Civil and Environmental Engineering, Imperial College London, South Kensington Campus, London, SW7 2AZ, UK

[2] Skanska UK, 1 Hercules Way, Leavesden, WD25 7GS, UK

[3] Net Zero Centre, Department of Engineering, King's College London, Strand, London, WC2R 2LS, UK

* Corresponding Author. Email: [a] sam.burdett@skanska.co.uk; [b] mohit.arora@kcl.ac.uk; [c] r.myers@imperial.ac.uk.

S1. Sustainable materials selection with emerging structural materials

An Excel spreadsheet titled Supplementary Dataset 1 is provided as electronic supplementary information that contains the data collected for this study and calculations for the materials selection case studies presented in the main text.

S2. Material Indices for structural components

Table S1. Examples of Ashby's materials indices for structural components (adapted from Ashby, 2017, Chapters 4-5 and Appendix B).

Component and constraints	Objective	Material Index (M; Maximise for optimal)	Dimension	Example insight
Tie in tension, stiffness-limited	Minimum mass	$E\ \rho^{-1}$	m^2s^{-2}	Favours high-stiffness, low density materials like composites.
Beam in bending, stiffness limited	Minimum mass	$E^{1/2}\ \rho^{-1}$	$kg^{-1/2}m^{5/2}s^{-1}$	Timber or aluminium efficient for light, stiff beams.
Beam in bending, strength-limited	Minimum mass	$\sigma^{2/3}\ \rho^{-1}$	$kg^{-1/3}m^{7/3}s^{-4/3}$	High-strength steels or reinforced concrete for failure-resistant design.
Column in compression, buckling (stiffness-limited)	Minimum mass	$E^{1/2}\ \rho^{-1}$	$kg^{-1/2}m^{5/3}s^{-1}$	Like beams; low-density woods for short columns.
Panel in bending, stiffness-limited	Minimum mass	$E^{1/3}\ \rho^{-1}$	$kg^{-2/3}m^{8/3}s^{-2/3}$	For floors/slabs; foams or sandwiches for ultra-light applications.

N.B. Indices can be extended for eco-design by dividing by EC (e.g., $E^{1/2} \rho^{-1} EC^{-1}$ for low-carbon, stiff beams).
